# Commentary: Phase I Trial of Carboplatin and Gemcitabine Chemotherapy and Stereotactic Ablative Radiosurgery for the Palliative Treatment of Persistent or Recurrent Gynecologic Cancer

**DOI:** 10.3389/fonc.2016.00263

**Published:** 2016-12-22

**Authors:** Charles A. Kunos

**Affiliations:** ^1^Cancer Therapy Evaluation Program, National Cancer Institute, Bethesda, MD, USA

**Keywords:** women’s cancer, radiation oncology, breast cancer, ovarian cancer, uterine cancer, cervix cancer, stereotactic body radiation therapy

Women diagnosed with breast or gynecological cancers seeking radiotherapy have not acted like typical health care consumers—they value considerably their initial oncologist’s treatment recommendations and put forward a general reluctance to deviate from these agreed initial approaches to care under early phase clinical trials evaluating new radiation-agent combinations (1) or hypofractionated radiotherapy (2, 3). Many female radiotherapy consumers now enter radiation oncology clinics as women who are “cancer survivors” knowledgeable of, and treated by, gainful biologic plus cytotoxic chemotherapeutic agents, and therefore, anticipate state-of-the-art radiotherapy. Fortunately, these female consumers have brand-new radiotherapy options, marketed in part by (a) better preclinical radiobiology, which more and more scrutinizes gains from radiation-agent combinations, (b) better clinical evidence from early phase clinical trials, extolling leading-edge radiation-agent approaches to cancer care, (c) better health care plans, alerting consumers to best clinical practices for radiotherapy length of care and shared insurer-insured financial responsibility, and (d) better home-town service, where local radiation oncologists provide first-rate radiotherapy cancer care.

But, some consumer myths need challenge, including that ovarian, uterine, and uterine cervix cancers are increasingly rare, are managed effectively without appreciation of molecular classification and are treated best by existing cancer stage-directed therapies.

Ovarian, uterine, and uterine cervix cancers in 2016 add up to be the third most common group of cancers in American women (4) but collectively are understudied and are underfunded in overall cancer research. For the year 2015, the United States National Institutes of Health (NIH) spent $269 million combined for ovarian, uterine, and uterine cervix cancer research, a sum only 40% of the $674 million spent for breast cancer research alone (5). There is an increasing unmet scientific gap in basic radiobiological cancer research to prime early or late phase clinical trials testing novel radiation-agent combinations for the treatment of gynecological cancers. It is imperative that such trials be guided by clinically relevant preclinical experiments that inform radiation-agent dose, schedule, exposure, and therapeutic effect for purposeful design of next-step clinical trials (6). Only then with such data in-hand could radiation-agent combinations, showing proof-of-target biomarker modification at clinically relevant radiation-agent dose and corresponding antitumor response, be prioritized for cancer care consumers seeking modern radiotherapy.

With fierce oncology market competition and limited resources, clinical trialist thought leaders must invest wisely in appealing radiotherapy cancer research, focusing on molecularly driven, safe, and practical radiation-agent combinations. Most of that gainful investment has been measured by early phase clinical trials that follow a 3 + 3 patient cohort design (7), recognizing trial priorities for participant wellbeing, principled conduct, and minimized interruption (8). Anecdotal evidence indicates that 3 + 3 designed radiation-agent trials might be most attractive if radiation course duration was condensed (e.g., 25-day pelvic radiation course shortened to 3 days) or if intensified radiotherapy was co-administered with less toxic administered doses of a molecularly targeted radiation/chemotherapy-sensitizing drug (e.g., gemcitabine) or conventional anticancer drug (e.g., carboplatin), as was done in the 12-patient referent 3 + 3 designed trial above (1). But sometimes, cancer care providers need to understand which trial service matters most (e.g., shortened radiotherapy course) to consumers in a particular disease setting and study closely that feature that aligns best with trial agreeableness. In the referent 3 + 3 designed trial above, there might have been a missed chance to use an accelerated titration designed trial (9)—where only one patient would have been accrued per cohort until one patient manifested a predefined dose-limiting toxic effect or two sequential patients experienced grade 2 toxic effects. When either of these two conditions are met, patient expansion occurs in that cohort for a traditional 3 + 3 design. Looking back, an accelerated titration design might have halved the 22-month accrual period in the above referent trial (1).

Women with breast or gynecological cancers are increasingly self-directed and are actively searching for best radiation services that optimally reduce radiation-related adverse events better than existing therapies. For this particular type of consumer, quality radiation service must be a beginning step (8). The movement away from existing radiation technology to less toxic machinery like intensity-modulated radiation therapy (IMRT) or stereotactic ablative radiosurgery (SABR) has altered radiation-related adverse event rates both in and out of clinical trials. Consumer demand has been met, but IMRT and SABR technology must be integrated slowly into cancer research. Quality radiation service for IMRT and SABR lags behind quality assurance, even in clinical trials (8). Until the quality service-assurance gap narrows, the mixing of radiation platforms in early phase radiotherapy cancer trial research should be avoided so that vital radiation-agent interactions could not be masked by overrepresented or by underrepresented radiation technology in an early phase cancer trial. While consumers have used—and will continue to utilize—radiotherapy proxies to demand treatment for best radiation service, it remains debated whether early phase radiotherapy trials should meet consumers where their demands are, or rather, whether to interrogate radiation technologies in stepwise early phase clinical trials.

Given this context, the articles in this *Frontiers in Oncology Research Topic* address open questions pertaining breast and gynecological cancer epidemiology, personalized medicine, and innovative therapeutic interventions.

## Author Contributions

Conception: CK; writing: CK; review and approval: CK.

## Conflict of Interest Statement

The author declares that the research was conducted in the absence of any commercial or financial relationships that could be construed as a potential conflict of interest.
